# Testing Lab-on-a-Chip Technology for Culturing Human Melanoma Cells under Simulated Microgravity

**DOI:** 10.3390/cancers13030402

**Published:** 2021-01-22

**Authors:** Dawid Przystupski, Agata Górska, Olga Michel, Agnieszka Podwin, Patrycja Śniadek, Radosław Łapczyński, Jolanta Saczko, Julita Kulbacka

**Affiliations:** 1 Department of Paediatric Bone Marrow Transplantation, Oncology and Haematology, Wroclaw Medical University, Borowska 213, 50-556 Wroclaw, Poland; dawid.przystupski@student.umed.wroc.pl; 2 Department of Molecular and Cellular Biology, Wroclaw Medical University, Borowska 211A, 50-556 Wroclaw, Poland; agata.d.gorska@gmail.com (A.G.); jolanta.saczko@umed.wroc.pl (J.S.); julita.kulbacka@umed.wroc.pl (J.K.); 3 Department of Cell Pathology, Faculty of Biotechnology, University of Wroclaw, Joliot-Curie 14a, 50-383 Wroclaw, Poland; 4 Faculty of Microsystem Electronics and Photonics, Wrocław University of Science and Technology, 50-370 Wrocław, Poland; agnieszka.podwin@pwr.edu.pl (A.P.); patrycja.sniadek@pwr.edu.pl (P.Ś.); 5 SatRevolution S.A, Stabłowicka 147, 54-066 Wroclaw, Poland; r.lapczynski@satrevolution.com

**Keywords:** microgravity, multidrug resistance, cisplatin, melanoma, cell death, LOC

## Abstract

**Simple Summary:**

The main aim of this study was to investigate whether all-glass Lab-on-a-Chip (LOC) platforms can be applied to cancer cell research performed under simulated microgravity. For this purpose, we designed and constructed a 3D-clinostat—a device that allows us to investigate the effect of simulated microgravity (*sµg*) in biological studies. We used human keratinocytes HaCaT and skin melanoma A375 cells cultured on LOCs as a research model. Preliminary analyses included optimization of LOCs structure and evaluation of their biocompatibility. For both cell lines, we demonstrated that LOCs can be successfully implemented in microgravity research. These results are a good base to conduct further research on the possible application of LOCs systems in cancer research in space, especially for microgravity studies.

**Abstract:**

The dynamic development of the space industry makes space flights more accessible and opens up new opportunities for biological research to better understand cell physiology under real microgravity. Whereas specialized studies in space remain out of our reach, preliminary experiments can be performed on Earth under simulated microgravity (*sµg*). Based on this concept, we used a 3D-clinostat (3D-C) to analyze the effect of short exposure to *sµg* on human keratinocytes HaCaT and melanoma cells A375 cultured on all-glass Lab-on-a-Chip (LOC). Our preliminary studies included viability evaluation, mitochondrial and caspase activity, and proliferation assay, enabling us to determine the effect of *sµg* on human cells. By comparing the results concerning cells cultured on LOCs and standard culture dishes, we were able to confirm the biocompatibility of all-glass LOCs and their potential application in microgravity research on selected human cell lines. Our studies revealed that HaCaT and A375 cells are susceptible to simulated microgravity; however, we observed an increased caspase activity and a decrease of proliferation in cancer cells cultured on LOCs in comparison to standard cell cultures. These results are an excellent basis to conduct further research on the possible application of LOCs systems in cancer research in space.

## 1. Introduction

### 1.1. Description of Lab-on-Chips

The term “Lab-on-a-Chip” (LOC) is generally understood as an autonomic or semi-autonomic experimental platform providing lab-scale research capabilities within miniaturized devices. With a size fitting within a few square centimeters combined with microfluidic systems capable of handling pico- to microliters of fluid volume, the use of LOCs drastically reduces the volume of samples, consumption of reagents, and time of analysis, thus lowering research costs. In the past decade, LOCs have found numerous biomedical applications, including integrated bioanalyses [1,2], metabolomics [3], drug discovery and delivery systems [4,5,6,7], cell analysis [8,9], tissue and organ physiology and disease modeling [10,11,12], and personalized medicine [11,13,14].

Typically, most LOC devices are disposable, fabricated using polydimethylsiloxane (PDMS) replica molding techniques [15,16,17]. These methods are considered fast, relatively simple, with moderate biocompatibility and acceptable transparency of the obtained structures. Nevertheless, according to the latest literature reports [18,19,20], some unfavorable influence of this material on biological samples—concerning contamination and/or depletion of cell culturing medium—has been observed, which can be especially critical in the case of long-term, cellular-based experimentation.

### 1.2. Advantages of LOC Technology

LOC instrumentation is usually used for pioneering “first-time” investigations; thus, uncertain biocompatibility is undesirable. For this reason, other materials (e.g., polystyrene or glass) are becoming increasingly popular with microfluidics because of their well-known and verified interactions with biological samples [20,21,22,23]. These and other matters (no time degradation, absolute chemical and biological compatibility, and excellent optical properties in visible spectrum range) can be essential in the context of the novel scientific approaches aiming at sophisticated biomedical research. Lately, LOCs have been gaining more attention as a promising tool in astrobiological experiments, with an emphasis on those performed in space aboard the International Space Station (ISS) [24,25,26] or launched on satellites [27]. Several studies proposed implementing LOCs in other research systems, including ground-based facilities designed for altered gravity investigations [28,29,30] or parabolic flight experiments [31]. One of the main issues of that kind of research lies in the limited feasibility of manual handling within the experimental unit, which is why remotely controlled hardware is strongly preferred. Another crucial feature is the compact size of the experiment. While sounding rockets and parabolic flights allow to conduct quite sizeable experiments exceeding the size of 10 cm × 10 cm × 10 cm cubic units, it remains a basic unit of the research nanosatellites CubeSat [32,33], as well as the ICE Cubes Service-cubic experiments placed on the ISS to carry our investigations in real microgravity [34].

### 1.3. Skin Melanoma and Space Research

Gravity variations have been shown to remarkably influence growth and biological processes of malignant cancer cells related to cell death and cell cycle arrest, drug resistance, angiogenesis, and cytokine secretion [35,36,37,38,39,40]. Due to the relationship between harmful irradiation (especially presented in space) and skin malignancies, melanoma represents a valuable experimental model for space research. However, the effect of microgravity on human melanoma cells has been barely investigated. Thorough studies may provide us with a brand-new information concerning the interactions between radiation-triggered cancers and microgravity exposure. Although the effectiveness of systemic therapy of melanoma is increasing, innovative adjuvant therapies are not free of side effects. More importantly, an increase of the number of melanoma patients and deaths caused by this malignancy has been observed. The occurrence of multidrug resistance and high metastatic potential make melanoma serious medical problem. Complete remissions are rare and the 5-year survival rate is still relatively low [41]. Taking into consideration abovementioned findings, we believe that gravity-related experiments concerning melanoma cells may improve our understanding of cancer biology and be useful in detecting interesting target pathways and proteins for future cancer treatment [42]. 

### 1.4. Microgravity Experiments

Although ground-based facilities for altered gravity research are generally less demanding in terms of size and handling, there is still a great need to establish new, optimized solutions. The most popular devices for microgravity simulation enable us to change the position of an accommodated biological samples in two- or three-dimensional space (clinostats) [43] with an option to be randomly altered by dedicated software (random positioning machine, RPM) [44]. Ideally, cell culture dishes in those facilities should be completely filled with medium devoid of air bubbles to avoid unspecific results generated by shear stress during the microgravity simulation [45,46]. Dealing with shear stress is time-consuming and impractical due to the troublesome procedure of sealing the dishes and it generates considerable costs resulting from volumes of medium required for filling the dish completely. A well-optimized system utilizing LOCs stands a chance to eliminate those issues. More importantly, there is a market discrepancy between reported cell response to altered gravity when referred to various research systems, including differences between real and simulated microgravity [47]. With that in mind, further comparative studies are urgently required, and LOCs seem to be a promising consensual solution suitable for most research systems.

Here, we investigated the technological and material biocompatibility of newly designed all-glass LOCs for culturing human cancer and normal cells in vitro during the experiments in simulated microgravity conditions. This paper is a preliminary attempt to establish new experimental system based on LOC technology and optimize of its use in 3D-clinostats. 

## 2. Results

### 2.1. Biocompatibility of LOCs

**Figure 1:**
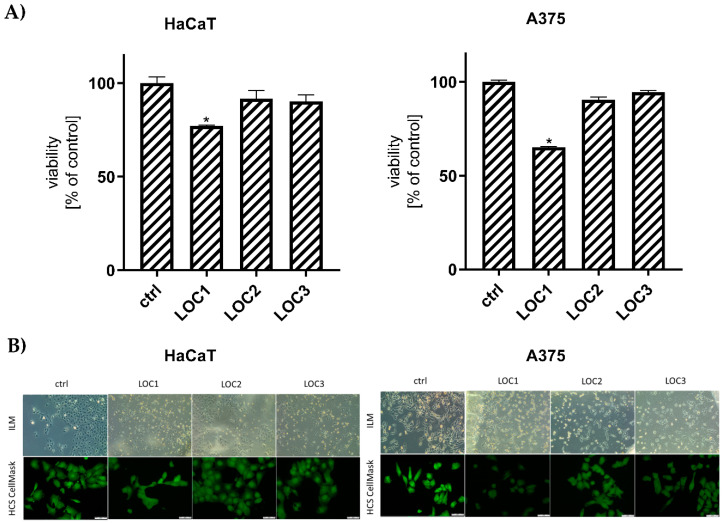
Evaluation of HaCaT and A375 cells viability (**A**) and morphology (**B**) after 24 h culturing on various Lab-on-a-Chip (LOC) (ILM: inverted light microscope); scale bars: 50 μm. * Statistically significant differences in comparison to control cells (*p* ≤ 0.05).

We performed viability and morphology analyses to determine the optimal LOCs for the selected cancer cell lines. Our studies revealed a decreased viability of HaCaT and A375 cells cultured on LOC1 (Figure 1A, 77.18%, and 65.14%, respectively). On the other hand, the viability of the cells remained unchanged both for LOC2 and LOC3. Moreover, we noted perfect agreement between PrestoBlue assay and High Content Screening (HCS) CellMask, indicating the presence of cells with a proper morphology on LOC2 and LOC3 (Figure 1B). Based on these findings, only LOC2 and LOC3 were recommended for the described research, and LOC1 was excluded from further experiments; in microgravity studies, we used only LOC3. 

### 2.2. Simulated Microgravity Experiments.

#### 2.2.1. Viability and Mitochondrial Activity

**Figure 2:**
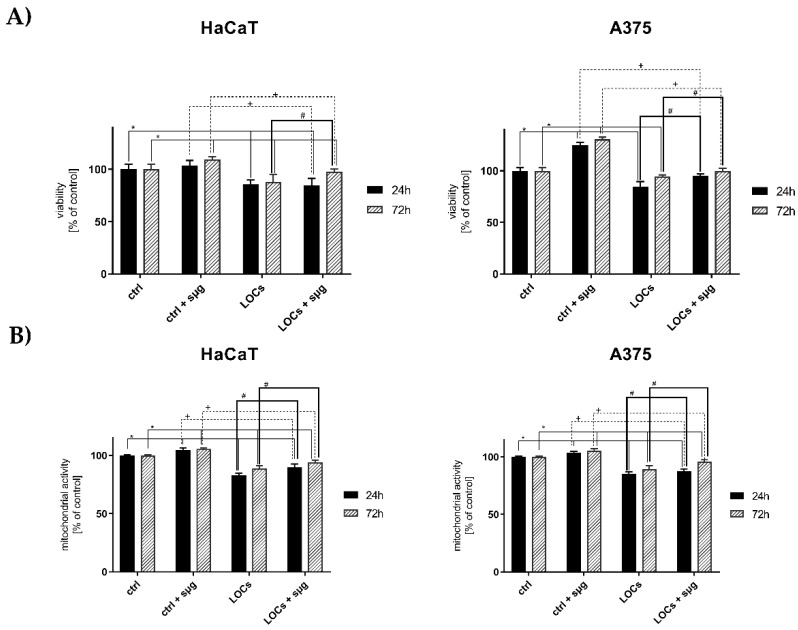
Evaluation of HaCaT and A375 cells viability (**A**) and mitochondrial activity (**B**) after the exposure to simulated microgravity. Statistically significant differences in comparison to *ctrl* cells: *, *ctrl + sμg* cells: +, and LOC cells: # (*p* ≤ 0.05).

Following the clinorotation, we observed a slight increase of HaCaT and A375 cell viability exposed to simulated microgravity (Figure 2A). The increase was more prominent for cancer cells (130% in 72 h). However, both HaCaT and A375 cells cultured on LOCs displayed a decreased viability compared to the cells cultured on Petri dishes during the clinorotation.

The mitochondrial activity of the cells cultured on Petri dishes remained unchanged (~100%) for 1 g and *sμm* groups in 24 and 72 h. At the same time, the mitochondrial functioning considerably decreased when the clinorotated cells were cultured on LOCs (Figure 2B, approx. 85–90% in 72 h). As for viability, the mitochondrial functioning intensified after the exposure to simulated microgravity.

#### 2.2.2. Caspase Activity

**Figure 3:**
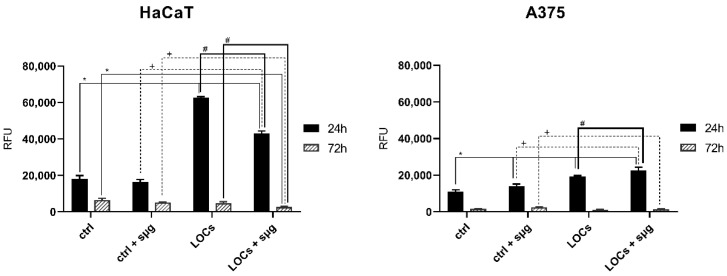
Caspase activity evaluation of HaCaT and A375 cells after the exposure to simulated microgravity; (RFU—relative fluorescence units). Statistically significant differences in comparison to *ctrl* cells: *, *ctrl + sμg* cells: +, and LOC cells: # (*p* ≤ 0.05).

According to our research, the exposure to simulated microgravity significantly increased the caspase 3/7 activity of the A375 melanoma cells 24 h after the clinorotation (Figure 3). Interestingly, we did not detect this phenomenon in HaCaT cells. Conversely, we observed an increased caspase activity 24 h after the clinorotation in the cells previously cultured on LOCs-especially HaCaT cells. That tendency was most evident in the HaCaT cells.

#### 2.2.3. Proliferation

**Figure 4:**
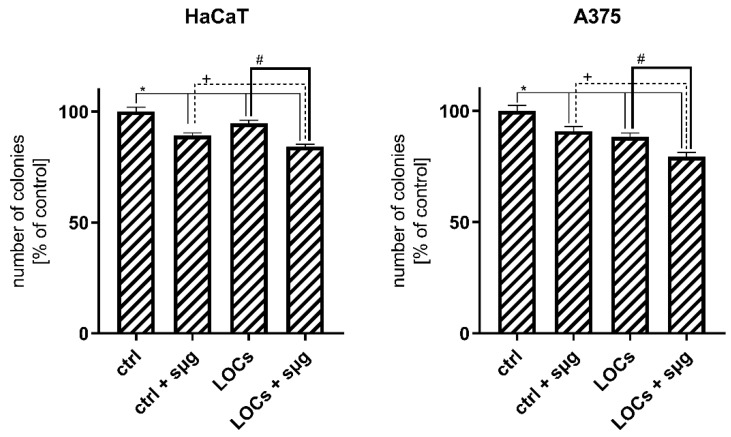
Number of colonies (% of control cells) after the exposure to simulated microgravity and 7-day incubation of HaCaT and A375 cells (* *p* ≤ 0.05; data are presented as the mean amount of counted colonies). Statistically significant differences in comparison to *ctrl* cells: *, *ctrl + sμg* cells: +, and LOC cells: # (*p* ≤ 0.05).

The clonogenic assay confirmed the inhibitory impact of simulated microgravity on cell proliferation (Figure 4). The reduced number of colonies between the *1 g* and *sμm* cells indicates the slight cytotoxic properties of *sµg*. Moreover, this assay demonstrated the decreased proliferation in cells cultured on LOCs and seeded on Petri dishes during the clinorotation. On the contrary, we observed fewer colonies when A375 cells were subjected to *sµg* and cultured on LOCs; however, the tendency was the same in control samples seeded on Petri dishes.

#### 2.2.4. Morphology

**Figure 5:**
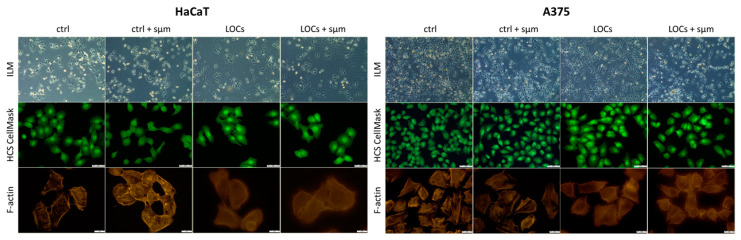
The representative photographs of the morphology of HaCaT and A375 cells exposed to simulated microgravity (ILM); scale bars: 50 μm for HCS CellMask and 20 μm for F-actin.

Fluorescence staining revealed considerable differences in the cell morphology after the clinorotation for 2 h (Figure 5). After *sµg*, the cells were shrunken and round shaped. Inverted light microscopy and HCS CellMask staining revealed the altered cell shape, presence of membrane blebbing and lamellipodia, lack of filopodia, and the presence of stress fibers. Cytoskeleton staining revealed distinct reorganization of F-actin fibers, which were disrupted and accumulated in peripheral parts of cells-both in HaCaT and A375 cells. One of the technical issues we had to deal with during the experiments with LOCs was photographing cells under high magnification. Because of LOCs thickness and technical limitations of camera, we could not obtain photographs with sharp outlines of the cells.

## 3. Discussion

In this paper we described the possible application of novel all-glass LOCs in short-term *sμg* experiments on human cells in vitro, investigating the potential shortcomings and advantages compared to the classical approach. The morphology of clinoratated cells cultured on LOCs resembled the morphology of similarly treated cells cultured in standard conditions, demonstrating alterations well-described in the literature: the altered, rounded cells, peripheral cytoskeleton accumulation, or membrane blebbing [48,49] caused by cytoskeletal rearrangements [50,51,52,53,54]. Going further, we observed the specific influence of clinorotation on the cells cultured on LOCs, consistent with literature reports concerning the impact of *sμg* and *μg* on the cell death regulation [55,56] and reduced proliferation [40,56,57,58,59,60,61]. In our other microgravity research concerning short-time clinorotation, we noticed the altered functioning of cancer cells 72 h after *sμg* exposure, especially in the case of cell death and cell cycle. However, we did not observe such phenomenon for HaCaT and A375 cells. Well-maintained cell viability observed within the control group subjected to *sμg* has been previously reported for short-term exposure to real *μg* during the parabolic flights [62]. Analogous tendencies were observed within LOC groups as well, but lowered accordingly compared to controls, suggesting an unresolved issue related to the cellular stress or assay methodology. The observed alterations are consistent with the initially increased caspase activity in cells cultured on LOCs. On the other hand, no signs of apoptosis within the control group corresponds well with altered gravity cellular response reported in other studies [63]. Further data collection is needed to eliminate those shortcomings. One possible explanation may be associated with a slightly disrupted ratio of nutrients and metabolites related to the limited volume of the culture medium. While not visible in normal conditions during the biocompatibility tests, the additive effect of *sμg* stress response might enhance its cytotoxic effect. Designing a culture chamber in LOCs requires the perfect balance between the largest possible culturing surface and optimal width facilitating diffusion. Thus, the amount of accessible supplies is limited by the volume of the culture areas [64]. Furthermore, these preliminary results have their limitations due to the restricted volume of medium available during the experiment. When the growth medium is not being replaced inside the culture chamber, cells consume glucose and oxygen leading to their decreased concentration [65]. Moreover, limited diffusion of oxygen causes the increased production of lactate, which lowers the pH of the medium [66]. Summing up, if cells are not provided with fresh nutrients, the metabolic processes are inhibited as observed for LOC1 [67,68]. Because we did not observe significant metabolic stress in cells cultured on LOC2 and LOC3 for 2 h during the clinorotation, we claim that constant administration of fresh medium is not necessary in short-time investigations. Research into solving this issue is already underway, since our all-glass LOCs are ultimately dedicated to the automated microfluidic flow of the medium.

Our study encourages the use of LOCs in examining the cellular response to simulated microgravity conditions. Looking forward, the presented LOCs could be placed in a portable bioreactor with a microfluidic system and automated administration of reagents while the all-glass transparent structure of our LOCs facilitates the use of cameras and microsensors [1,69]. Overall, the size of that kind of assembly would not exceed 1 U. The advantage of this solution is the possibility that any further development of this technology may find applications in remotely controlled experiments in real microgravity, e.g., parabolic flights, sounding rockets or nanosatellites, which could be a promising solution for the comparative analyses of the experiments in ground-based facilities. 

## 4. Materials and Methods

### 4.1. Cell Culture Maintenance

All experiments were performed on two human cell lines: immortal keratinocytes, HaCaT, and malignant melanoma, A375 (ATCC, Manassas, VA, USA). Cell cultures were maintained in a CO_2_ incubator at 37 °C in Dulbecco’s Modified Eagle’s Medium (Sigma-Aldrich, St. Louis, MO, USA) supplemented with 2 mM ultraglutamine (Lonza, Basel, Switzerland), 10% fetal bovine serum (Atlanta Biologicals, Norcross, GA, USA), 1% MEM vitamin solution (100×, Sigma), and antibiotics: 100 IU/mL penicillin, and 0.1 mg/mL streptomycin (Gibco, Gaithersburgh, MD, USA).

### 4.2. LOCs Specification

**Figure 6:**
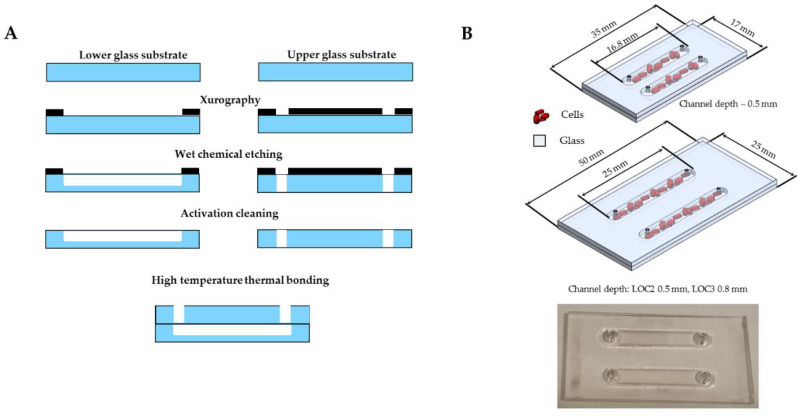
Fabrication of all-glass LOCs (cross-section view), technology flow (**A**); the structure of the prepared LOCs (**B**).

All-glass LOCs were fabricated utilizing standard glass micromachining processes, i.e., xurography, wet chemical etching and high temperature fusion bonding, Figure 6A [70,71]. Firstly, two laboratory autoclaved borosilicate glass substrates were covered with special hydrogen fluoride (HF)-resistive foil (Avery Dennison Graphics Solutions, USA) containing micropatterns of microchannels with CNC laser-cut holes. Next, the substrates were chemically etched in the solution of 40% HF: 69% HNO_3_ (10:1 *v*/*v*) for a specified time (etching rate: 3 µm/min). After this step, the activation cleaning of the substrates was employed prior to bonding. This procedure encompassed washing with trichloroethylene, acetone, IPA, and submersion in Piranha solution (98% H_2_SO_4_: 30% H_2_O_2_, 3:1 *v*/*v*) for approximately 10 min. As a final fabrication step, the substrates were carefully cleaned with deionized water, positioned and bonded thermally in a furnace (650 °C). Three different LOCs were developed for the experiments: one the size of 16.8 mm × 3.5 mm with 500 µm microchannel depth (LOC1), as well as two 25 mm × 3.5 mm containing microchannel of 500 µm (LOC2) and 800 µm (LOC3) depth. Our all-glass LOCs are reusable and, after careful cleaning and disinfection, they can be used for cell cultivation analyses many times. All LOCs were investigated for their biocompatibility to select the most optimal chips for HaCaT and A375 cells, as described below.

### 4.3. Cultures On-Chip

For experiments, the cells were detached with TrypLE™ Express Enzyme (ThermoFisher Scientific, Waltham, MA, USA), centrifuged and resuspended in a CO_2_ independent medium (Thermo Fisher Scientific). Then, the cells were seeded in a chip chamber at ~1 × 10^6^ cells/mL. Cultures were observed and photographed daily using a DMi1 inverted microscope (Leica, Wetzlar, Germany) equipped with LAS X imaging software (Leica).

Prior to the microgravity studies we performed pilot experiments assessing biocompatibility of the LOCs differing in structure (Figure 6B), which included: (i) evaluation of the viability of cells cultured on LOCs with PrestoBlue assay (described below), and (ii) analysis of the morphology of the cells seeded on LOCs using inverted light and fluorescence microscopes (HCS CellMask™ Green Stain staining-described below)-both assays were evaluated 24 h after culturing the HaCaT/A375 cells on LOCs.

### 4.4. Simulated Microgravity Experiments

**Figure 7:**
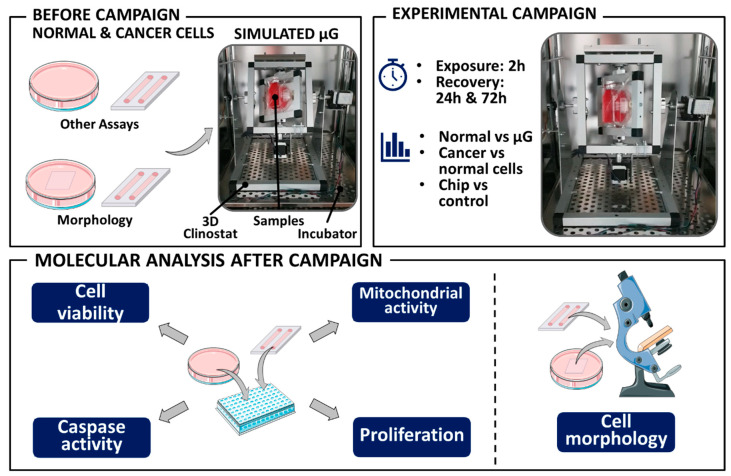
The schematic representation of the procedure of simulated microgravity experiment.

To examine the effect of simulated microgravity (*sμm*) on HaCaT and A375 cells, a 3D-clinostat (3D-C) was developed by engineers from Wroclaw University of Science and Technology. To provide microgravity conditions, the biological samples were placed in the 3D-clinostat and rotated along two independent axes at constant speeds and directions relative to the gravity vector, eliminating the effect of gravity [43]. The base, outer, and inner parts are made of aluminum rectangular profiles perforated with a Computerized Numerical Control (CNC) machine. The main frame is made of stainless steel plate with laser-cut perforation. The frame, outer, and inner parts are connected with aluminum pipes in which the electrical wires are routed. The outer and inner parts rotate independently. Two step motors provide the rotational movement with speed from 0.1 rpm to 60 rpm. The motors are controlled by a step motor controller DRV8835 build on a microcontroller. The user can switch rotational speed with a knob. Rotating elements were balanced to avoid vibrations. The rotation speed was set at 10 rpm (60°/sec; 1.05 rad/sec, Figure 7). 

For *sμm* experiments, cells were seeded in the selected LOCs. After an hour, the LOCs with cells were put in Petri dishes filled with growth medium to provide diffusion of nutrients etc. After 24 h, the growth medium was replaced and the chips were carefully covered with parafilm without leaving any air bubbles beneath it. In our research we decided to use the smallest growth dishes with rounded-shaped walls to reduce the shear stress accompanying the clinorotation. The cells cultured on 35 mm Petri dishes (3 × 10^4^ cells/cm^2^) and 35 mm imaging dishes (µ-Dish 35 mm, Ibidi, Germany) were used as control samples (*ctrl*) prepared in the same way as LOCs. The exposure time in *sμm* was 2 h. Taking into consideration the fact that we used in the *sμm* experiment 35 mm dishes for culturing the cells, we can estimate the rotation radius approximately 18 mm and 12.5 mm for LOCs. Based on the study of Eiermann et al. (2013), the maximal residual accelerations varied between 0.024–0.036 g [72]. The samples were fixed on the clinostat in an incubator at 37 °C. The static control group (*ctrl*) was prepared the same way and located close to the 3D-C during the clinorotation to distinguish the effect of mechanical stress caused by vibrations of the 3D-C. After the experiment, all cells were rinsed with PBS three times, detached using TrypLE™ Express Enzyme and processed for further analyses. For viability, mitochondrial and caspase activity assays of the cells were seeded on 96-well plates (1.5 × 10^4^ cells/cm^2^) and incubated for 24 or 72 h. Additionally, the cells were plated on 6-well plates and incubated for 7 days to carry out the clonogenic assay (cell density seeding 15 cells/cm^2^). Part of the samples prepared for morphology and cytoskeleton analyses was fixed immediately after the experiment.

### 4.5. Viability

Cell viability was examined with a PrestoBlue™ Cell Viability Reagent (ThermoFisher, cat. No. A13262). For the viability testing of cells seeded on-chip, the growth medium was removed from the chip. The reagent was diluted 1:9 with a culture medium, and the solution was added to each chip channel. After 40 min of incubation at 37 °C, the solution was gently mixed with a pipette. Then 60 µL of the mixture was collected twice from each channel and disposed into the bottom of two wells of the black, 96-well plate with a transparent bottom. The cells were detached from the LOCs using TrypLE™ Express Enzyme and then seeded on a black, 96-well plate with a transparent bottom for microgravity experiments. After incubation, the growth medium was removed from the wells, and the reagent was diluted 1:9 with a culture medium, and 150 µL of the solution was added to each well. After 40 min of incubation at 37 °C, the solution was gently mixed with a pipette. The fluorescence was measured using a GloMax^®^ Discover Microplate Reader (Promega) with Green 520 nm excitation filter and 580–640 nm emission filter.

### 4.6. Mitochondrial Activity

MTS assay (CellTiter 96^®^ AQueous One Solution Cell Proliferation Assay; Promega, Madison, WI, USA) was performed to analyze mitochondrial function. Following the clinorotation, 20 μL of CellTiter 96^®^ AQueous One Solution Reagent was added to each well and the cells were incubated with the reagent for 2 h at 37 °C. The absorbance was measured at 490 nm using a microplate reader (GloMax Discovery, Promega). The results were expressed as the percentage of cells mitochondrial activity relative to untreated control cells (*ctrl*).

### 4.7. Measurements of Caspase 3/7 Activity

The Caspase-Glo^®^ 3/7 Assay System (Promega) was used to measure the activity of caspases 3 and 7 in cells treated with the use of simulated microgravity. Following the *sµg* exposure, cells were seeded on white-walled 96-well plates. After an appropriate incubation time, 100 μL of Caspase-Glo^®^ 3/7 Reagent was added to each well and the cells were incubated for an hour at room temperature with the reagent. Next, the luminescent signal was collected with the GloMax^®^ Discover Microplate Reader (Promega). The results were expressed as the percentage of cells caspase 3/7 activity relative to untreated control cells (*ctrl*).

### 4.8. Clonogenic Assay

The clonogenic assay was performed according to the procedure described previously [73]. Following the exposure to simulated microgravity, the cells were harvested from the chip using the TrypLE™ Express Enzyme, counted and then 15 cells/cm^2^ were seeded on 6-well culture plates. After 7 days of incubation, the cells were washed with Phosphate Buffered Saline (Sigma-Aldrich), fixed with 0.5% crystal violet (Sigma-Aldrich) in 4% paraformaldehyde (Sigma-Aldrich), then rinsed with tap water and left to dry. The colonies were photographed with a DMi1 inverted microscope (Leica) and then analyzed with the ImageJ software. The number of colonies was presented as percentage of non-treated control cells (ctrl).

### 4.9. Fluorescence Imaging

Some of the experiments were followed by fluorescence staining applied to the cells seeded on LOCs and the cells seeded on control glass according to producers’ protocols. The cell’s morphology was demonstrated using a HCS CellMask™ Green Stain (Thermo Fisher, cat. No. H32714). F-actin was visualized with an Alexa Fluor™ 546 Phalloidin (1:100 in PBS, Thermo Fisher; cat. No. A22283). The imaging was performed with an inverted microscope IX53 (Olympus, Tokyo, Japan) using the following excitation/emission filters: U-FBN (Narrow band blue excitation, 470-495/510 nm) for HCS CellMask and U-FGW (Wideband green excitation, 530-550 /575 nm) for Alexa Fluor™ 546 Phalloidin, respectively. Images were captured with the CellSens Imaging Software (Olympus). Cell morphology was assessed by two experimenters who independently analyzed and evaluated all collected samples from the particular groups (app. 100 cell from every slide) and compared them with “*ctrl*” group. 

### 4.10. Statistics

Two-way ANOVA determined statistical significance with Tukey’s post-hoc test within groups following a normal distribution. Samples were analyzed in four replications in three independent experiments. Differences within *p* values ≤ 0.05 were assumed to be statistically significant. The results were analyzed with the Microsoft Office Excel 2017 and GraphPad Prism 7.0 software (GraphPad Software, San Diego, California USA). The error bars presented on the graphs stand from the standard deviation calculated from three experiment replicates. For morphology analyses, approximately 100 cells were assessed.

## 5. Conclusions

In summary, this is the first in vitro study considering the investigation of the effect of simulated microgravity on human keratinocytes and skin melanoma cells cultured on all-glass LOCs. We devised a strategy for imaging living and fixed cells on the LOCs and successfully performed simple cellular assays to assess the cells’ response. Our experiments confirmed typical alterations in human cell physiology after a short-time exposure to simulated microgravity, namely the decrease of cell viability and mitochondrial activity, the increased activity of caspase activity, and reduced proliferation. These observations were also accompanied by altered cell morphology: the presence of stress fibers, membrane blebbing, and lamellipodia, as well as a lack of filopodia. On that basis, the described LOCs can be applied to various cell culturing experiments and supplemented with diverse sensors allowing for indirect analysis of biological properties of cells or microfluidic systems for real-time investigations and administration of reagents. Moreover, all-glass LOCs can be used for fluorescence microscopy; however, observations performed under higher magnification may be challenging, and the use of thinner chips may be necessary. Further research is needed to verify whether all-glass LOCs can be used in microgravity studies performed in space aboard the ISS or the CubeSat satellites.

## Data Availability

The data presented in this study are available on request from the corresponding author.
